# Single‐Cell Transcriptome Analysis Uncovers La Ribonucleoprotein 6 (LARP6) as a Dual Regulator of Proliferation and Immune Infiltration in Triple‐Negative Breast Cancer

**DOI:** 10.1111/jcmm.70709

**Published:** 2025-07-09

**Authors:** Feng Yuan, Gai Liang, Qu Zhang, Bo Luo, Jianhua Liu, Xinhong Wu

**Affiliations:** ^1^ Breast Cancer Center Hubei Cancer Hospital, Tongji Medical College, Huazhong University of Science and Technology, National Key Clinical Specialty Discipline Construction Program, Hubei Provincial Clinical Research Center for Breast Cancer, Wuhan Clinical Research Center for Breast Cancer Wuhan Hubei China; ^2^ Department of Radiotherapy Center Hubei Cancer Hospital, Tongji Medical College, Huazhong University of Science and Technology, National Key Clinical Specialty Discipline Construction Program, Hubei Provincial Clinical Research Center for Breast Cancer, Wuhan Clinical Research Center for Breast Cancer Wuhan Hubei China

**Keywords:** cell cycle, immune infiltration, LARP6, single‐cell RNA sequencing, triple‐negative breast cancer (TNBC)

## Abstract

Breast cancer is classified into multiple subtypes, including hormone receptor‐positive (oestrogen/progesterone receptor, ER/PR), HER2‐positive (human epidermal growth factor receptor 2), and triple‐negative breast cancer (TNBC). Among these, TNBC is more aggressive and susceptible to recurrence. The identification of novel TNBC‐specific markers is crucial for the development of advancing therapeutic approaches for this subtype. In our study, firstly we integrated single‐cell RNA sequencing data from more than 260,000 cells from previously published breast cancer datasets with ER‐positive, HER2‐positive and TNBC samples, determined the cell types based on the marker genes and identified the differentially expressed genes across various cell types between TNBC and ER/HER2‐positive cancers using pseudobulk analysis. Additionally, we conducted gene set enrichment analysis (GSEA) with the differentially expressed genes and identified 8 pathways which are consistent between the comparisons of TNBC/ER‐positive and TNBC/HER2‐positive. Furthermore, we found the shared gene, LARP6 (La Ribonucleoprotein 6) was significantly upregulated in TNBC compared to ER and HER2‐positive breast cancers. Also, the result from survival analysis revealed that the high LARP6 level significantly affected patient survival. At last, we found LARP6 was highly expressed in the TNBC cell line, and knockdown of LARP6 reduced cell proliferation, which was associated with the cell cycle alterations as determined by TriCycle analysis. Immune infiltration analysis further revealed that LARP6 expression correlates with distinct immune cell populations in the tumour microenvironment, suggesting its role beyond cancer cell intrinsic functions.

## 1 Introduction

Breast cancer represents an aggressive and prevalent malignancy affecting approximately 2.3 million newly diagnosed women annually worldwide [1, 2, 3]. Breast cancer is a heterogeneous disease based on molecular characteristics. The subtypes vary in gene expressions and phenotypes, and could be grouped into three main groups: hormone receptor‐positive (ER/PR‐positive), HER2‐positive, and triple‐negative breast cancer (TNBC) [4, 5]. TNBC subtype cancer cells lack the expression of oestrogen receptors, progesterone receptors, and HER2. TNBC represents 15%–20% of all breast cancer cases, exhibiting markedly clinical aggressive behaviour and high rates of recurrence and metastasis [6].

While targeting therapies have significantly improved the survival ratio for hormone receptor positive and HER2 positive breast cancer subtypes, the clinical management of TNBC subtype is still challenging because of the lack of specific targets and the aggressive behaviour of the cancer cells [7, 8]. The absence of targetable molecular results in the poor outcomes of the clinical management of TNBC cancer patients [9]. Therefore, uncovering new molecular targets specific to TNBC is crucial for developing more effective therapeutic strategies.

Recently, the advances in single‐cell RNA sequencing technology have provided us the insight into breast cancer heterogeneity at cellular resolution [10, 11, 12, 13, 14]. Single‐cell transcriptome analysis provides the capability to understand the interactions between each cell type inside the cancer tissue and reveal their molecular signatures. Several studies have already employed single‐cell RNA sequencing to examine global changes in breast heterogeneity and complex cellular ecosystems across different subtypes [14, 15]. However, because of the confounding effects of batch variation, data integration of different datasets prior to downstream analysis is still important.

The La‐related protein (LARP) family, including Genuine La, LARP1, LARP1b, LARP4, LARP4b, LARP6, and LARP7, has been suggested to play important roles in transcription and/or mRNA translation [16]. Among them, LARP6 has been implicated to be upregulated in basal‐like invasive ductal carcinomas of the breast, and ectopic expression of LARP6 in breast cancer cells enhances proliferation and invasion [17]. However, the role of LARP6 in TNBC pathogenesis compared with the pathogenesis of ER+ and HER2+ subtypes remains largely unexplored.

In this study, we attempt to identify novel TNBC specific markers by comprehensively integrating published single‐cell RNA sequencing datasets from more than 260,000 cells from ER+, HER2+, and TNBC subtypes. We conducted detailed cellular annotation, pseudobulk analysis for differentially expressed genes, and pathway enrichment analysis to distinguish genes and pathways unique to the TNBC subtype when compared to the ER+ and HER2+ subtypes separately. The results revealed LARP6 was highly upregulated in TNBC cancer cells compared to ER+ and HER2+ cancer cells, promoting us to investigate the role of LARP6 in TNBC progression. After down‐regulation of LARP6 in the TNBC cell line, the results from the cell counting kit‐8 (CCK‐8) assay and EdU staining suggested the proliferation of the cancer cells was significantly affected. The affected proliferation of cancer cells was associated with the down‐regulated cell cycle proteins, Cyclin B1 and CDK1. Furthermore, immune infiltration analysis demonstrated significant correlations between LARP6 expression and the immune infiltration of specific immune cell populations, suggesting a potential role for LARP6 in modulating the TNBC tumour microenvironment beyond its cell‐intrinsic functions. Taken together, we demonstrate that LARP6 serves as a dual regulator of proliferation and immune infiltration of TNBC cells instead of ER+ and HER2+ cancer cells, suggesting its potential to be the specific therapeutic target for the aggressive TNBC subtype.

## 2 Materials and Methods

### 2.1 Single‐Cell RNA Sequencing Integration and Annotation

We analysed single‐cell RNA sequencing data from previously published breast cancer datasets GSE161529 [15] and GSE176078 [14]. 17 ER+ samples, 6 HER2+ samples, and 4 TNBC samples were subsetted from GSE161529. 9 ER+ samples, 3 HER2+ samples, and 8 TNBC samples were subsetted from GSE161529. Samples in the same subtype were integrated using the method of CCAIntegration using Seurat v5.1.0 [18]. The integrated datasets were then processed for dimensionality reduction using UMAP. More than 260,000 cells were then annotated based on the expressions of the marker genes.

### 2.2 Pseudo‐Bulk Analysis and Pathway Enrichment

For the analysis of differential expression genes between different cancer subtypes, we employed pseudobulk analysis by aggregating the gene expression matrix within each cell type. Muscat was used to identify differentially expressed genes with the cutoffs of adjusted *p*‐value < 0.05 and |log2FoldChange| > 1.2 [19]. The R package EnhancedVolcano was used to visualise the differential expression genes. Gene Set Enrichment Analysis (GSEA) was conducted using clusterProfiler with the Gene Ontology biological process database. Enriched pathways with adjusted *p*‐value < 0.05 were considered significant. Network analysis of enriched pathways was also visualised using clusterProfiler [20].

### 2.3 Cell Cycle Analysis Using TriCycle

Cell cycle phase inference for the single‐cell RNA‐seq datasets was conducted using the TriCycle R package (version 1.14.0) [21]. The single‐cell RNA‐seq data were projected to a pre‐learned cell cycle space and the cell cycle position, which is between 0 and 2π, was estimated using the estimate_cycle_position function. Approximately, 0.5π is the start of the S stage, pi is the start of the G2M stage, 1.5π is the middle of the M stage, and 1.75π–0.25π is the G1/G0 stage. The cell cycle distribution of the cells across different sample groups was visualised using the plot_emb_circle_scale function and ggplot2 themes.

### 2.4 Survival Analysis

Kaplan–Meier analysis was performed by stratifying patients into high and low LARP6 expression groups based on median expression value using Kaplan–Meier Plotter [22, 23]. Patients were grouped into high and low LARP6 expression groups based on the median expression value (cutoff value: 211). The analysis included all patients regardless of follow‐up threshold, with censoring applied at the threshold. Hazard ratios (HR) with 95% confidence intervals and log‐rank *p*‐values were calculated to assess statistical significance. To ensure data quality, redundant samples were removed and biased arrays were excluded. The proportional hazards assumption was verified. The analysis included patients who received any endocrine therapy or chemotherapy.

### 2.5 Cell Culture and Transfection

MCF‐7 (ER+), SK‐BR‐3 (HER2+) and MDA‐MB‐231 (TNBC) cell lines were obtained from ATCC and cultured in Dulbecco's modified Eagle's medium (DMEM, Gibco, 31600‐083) supplemented with 3.5 g/L glucose, 1.5 g/L NaHCO_3_, 1% Penicillin–Streptomycin (Gibco, 15140148), and 10% fetal bovine serum (FBS, Gibco, 10270‐106) at 37°C with 5% CO2. For LARP6 knockdown, cells were transfected with LARP6‐specific siRNA (Santa Cruz Biotechnology, sc‐90245), siRNA‐resistant LARP6 group, or negative control siRNA using Lipofectamine 3000 (Invitrogen, L3000001) following the manufacturer's protocol.

### 2.6 Quantitative RT‐PCR

Total RNA was extracted using TRIzol reagent (Invitrogen, 15596026) and reverse transcribed using SuperScript IV (Invitrogen, 18090010). qRT‐PCR was performed using SYBR Green Master Mix (Applied Biosystems, 4309155) on an ABI 7500 system. GAPDH served as an internal control. The 2^−ΔΔCt^ method was used to calculate the relative expression of the genes. The R package, qPCRtools, was used to calculate and visualise the qPCR results [24]. Primers used for quantitative PCR were as follows: GAPDH_forward: 5′‐GGTCGGAGTCAACGGATTTG‐3′, GAPDH_reverse: 5′‐GGAAGATGGTGATGGGATTTC‐3′; LARP6_forward: 5′‐CCGCAAGTGTATGGATTATTC‐3′, LARP6_reverse: 5′‐CTCTGGTGTTGTCAGGAC‐3′.

### 2.7 Cell Counting Kit—8 Assay and Click‐iT EdU Cell Proliferation Assays

To quantitate the viable cell number in proliferation, we performed a cell counting kit‐8 assay according to the manufacturer's instructions (Sigma‐Aldrich, 96992). For Click‐iT EdU Cell Proliferation Assays, cells were incubated with 10 μM EdU (Invitrogen, C10634) for 2 h at 37°C, and then fixed using 4% paraformaldehyde (PFA) for 15 min at room temperature. Cells were then permeabilised using 0.3% Triton X‐100 in PBS for 10 min. EdU was detected using the Click‐iT EdU Alexa Fluor 647 Kit (Invitrogen, C10634) based on the description in the manufacturer's manual. Following EdU labeling and detection, nuclei were counterstained with DAPI (1:2000, Thermo Fisher Scientific, D3571) for 30 min at room temperature. Cells were mounted and visualised using a Leica TCS SP8 confocal laser scanning microscope equipped with a 20× air objective. Z‐stack images (1 μm step size) were acquired across multiple fields to ensure comprehensive spatial sampling. Image acquisition parameters—including laser power, detector gain, and pinhole diameter—were maintained consistently across all experimental conditions. Quantification of EdU incorporation was performed manually by an investigator blinded to the experimental conditions using ImageJ software. For each field, a minimum of 200 DAPI‐positive cells were counted, and the number of EdU‐positive cells within this population was determined based on nuclear Alexa Fluor 647 signal distinctly above background fluorescence. The proliferation rate was calculated as the ratio of EdU‐positive cells to total DAPI‐positive nuclei for each field. At least five non‐overlapping fields were analysed per replicate to ensure representative sampling.

### 2.8 Western Blot

Cultured cells were collected in RIPA lysis buffer (50 mM Tris–HCl pH 7.4, 150 mM NaCl, 1% NP‐40, 0.5% deoxycholate, 0.1% SDS) supplemented with protease inhibitor cocktail (Roche, 4693132001). Protein concentration was determined using the BCA Protein Assay Kit (Pierce, 23227). Equal amounts of protein (30 μg) were loaded into and separated using 10% SDS‐PAGE gels and transferred to PVDF membranes (Millipore, IPVH85R) at 200 mA for 2 h at 4°C.

Membranes were blocked with 5% non‐fat milk (VWR, MSPP‐M0841) in 1% TBST (TBS with 0.1% Tween‐20) for 1 h at room temperature, then incubated with primary antibodies overnight at 4°C: anti‐LARP6 (1:1000, Sigma‐Aldrich, SAB2104561), anti‐Cyclin B1 (1:2000, Abcam, 4138 T), anti‐CDK1 (1:800, Abcam, ab32094), and anti‐GAPDH (1:5000, Cell signalling, 2118 T). Wash the membrane for 3 times with 1% TBST; membranes were incubated with HRP‐conjugated secondary antibodies (1:5000, Cell Signalling Technology) for 2 h at room temperature. Protein bands were visualised using enhanced chemiluminescence reagent (ECL, Thermo Scientific, 32106) and detected using the ChemiDoc imaging system (Bio‐Rad).

### 2.9 Statistical Analysis

All experiments were conducted with three replicates. Data are presented as mean ± SD. Statistical analyses were performed using R‐4.4.2. Student's *t*‐test was used for two‐group comparisons. *p* < 0.05 was considered statistically significant. Different levels of statistical significance were defined as: *p < 0.05, **p < 0.01, and ***p < 0.001.

## 3 Results

### 3.1 Integration and Annotation of Single‐Cell RNA Sequencing Datasets of Breast Cancer Subtypes

**FIGURE 1:**
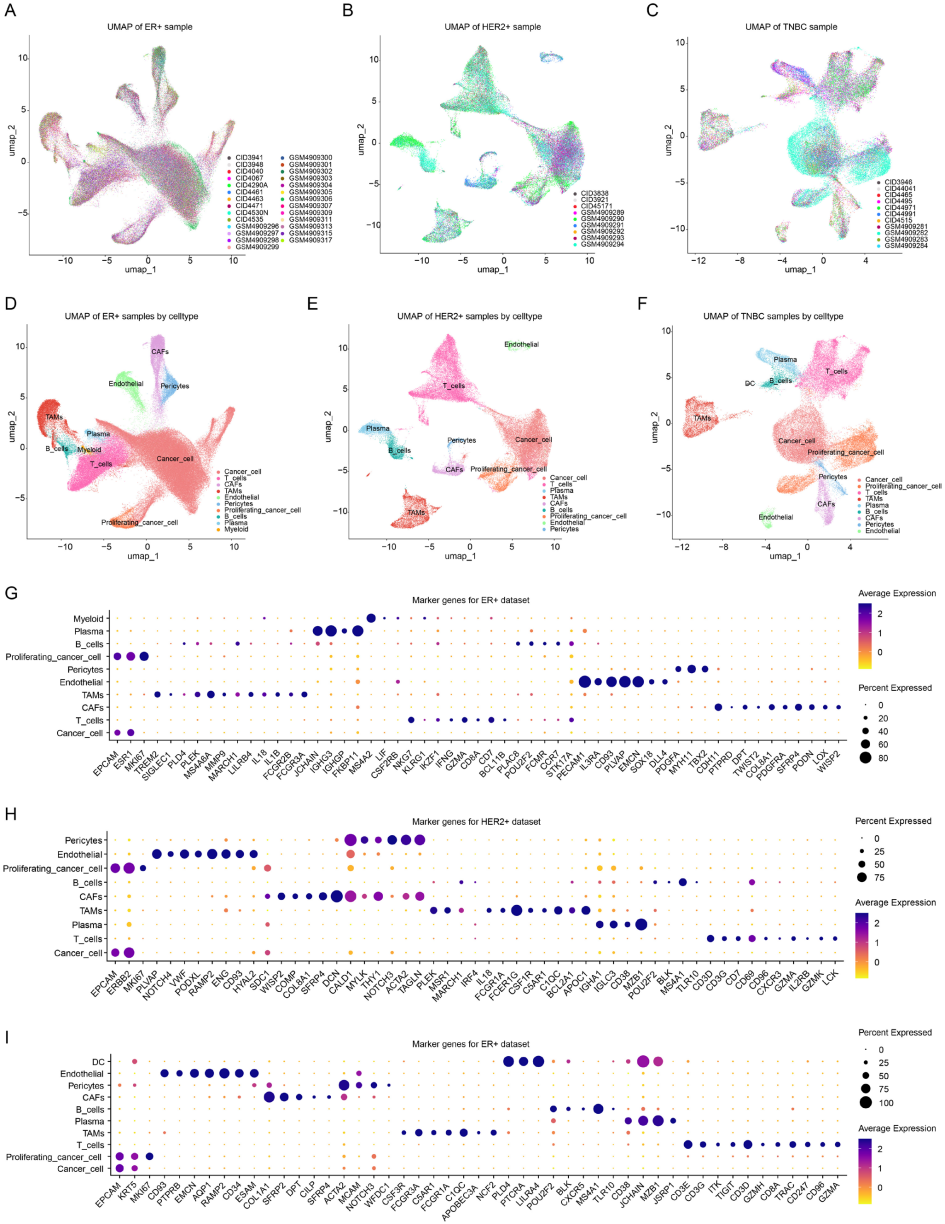
Integration and annotation of single‐cell RNA sequencing datasets of breast cancer subtypes. (A–C) UMAP visualisation of single‐cell transcriptomes from ER+ (A), HER2+ (B), and TNBC (C) samples, coloured by sample IDs. (D–F) UMAP visualisation of cell type annotations for the integrated ER+ (D), HER2+ (E), and TNBC (F) samples. (G–I) Dot plots showing the marker genes for each cell type in the integrated ER+ (G), HER2+ (H), and TNBC (I) datasets. Dot size stands for percentage of cells expressing the gene, and colour intensity indicates average expression level.

To identify the novel therapeutic targets specific to TNBC subtype, we integrated single‐cell RNA‐sequencing datasets from GSE161529 [15] and GSE176078 [14] and obtained more than 260,000 cells. 17 ER+ samples, 6 HER2+ samples and 4 TNBC samples were obtained from GSE161529. 9 ER+ samples, 3 HER2+ samples and 8 TNBC samples were obtained from GSE161529. We integrated the samples from different subtypes separately, and UMAP visualisation confirmed that all samples within each subtype were successfully integrated and distributed evenly (Figure 1A–C). Next, we performed downstream analysis and clustered the cells in each subtype based on the expressions of the marker genes. For ER+ subtype, we identified cancer cells, proliferating cancer cells, T cells, B cells, myeloid cells, endothelial cells, plasma, pericytes, tumour‐associated macrophages (TAMs), cancer‐associated fibroblasts (CAFs) (Figure 1D) based on the marker genes (Figure 1G). For HER2+ subtype, we identified cancer cells, proliferating cancer cells, T cells, B cells, endothelial cells, plasma, pericytes, tumour‐associated macrophages (TAMs), cancer‐associated fibroblasts (CAFs) (Figure 1E) based on the marker genes (Figure 1H). For TNBC subtype, we identified cancer cells, proliferating cancer cells, T cells, B cells, endothelial cells, plasma, pericytes, tumour‐associated macrophages (TAMs), cancer‐associated fibroblasts (CAFs) and dendritic cells (DCs) (Figure 1F) based on the marker genes (Figure 1I). Based on the defined annotation on the integrated dataset, we could then perform the differentially expressed gene analysis to reveal the transcriptomic changes among the subtypes of breast cancer.

### 3.2 Comparative Analysis of Differential Gene Expression Between TNBC Versus ER+ and HER2+ Subtypes

**FIGURE 2:**
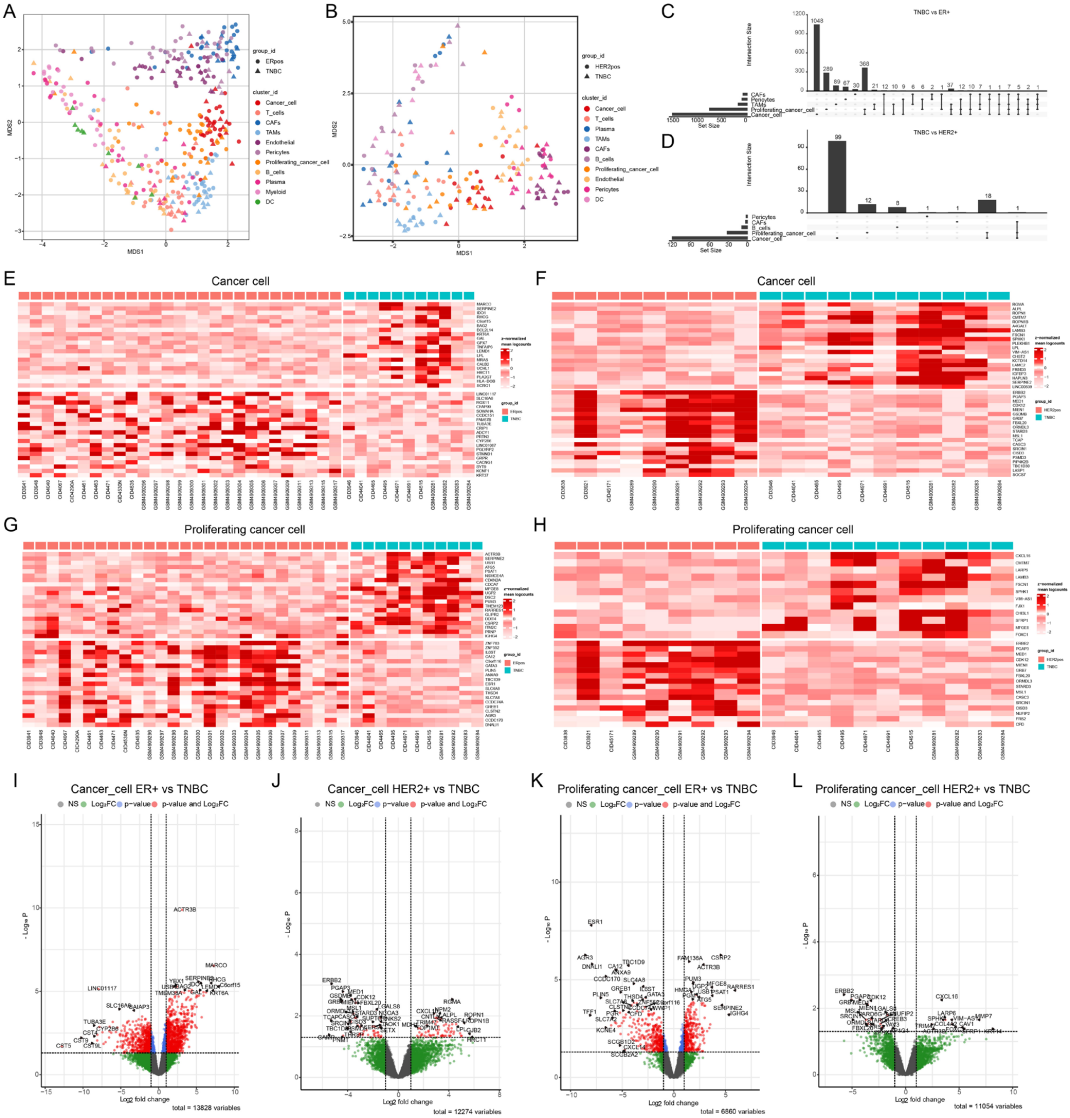
Comparative analysis of differential gene expression between TNBC versus ER+ and HER2+ subtypes. (A, B) MDS plots showing clustering of different cell types in TNBC vs. ER+ and TNBC vs. HER2+ comparisons. (C, D) UpSet plots showing the complex association between TNBC and ER+/HER2+ samples across cell types. (E–H) Heatmaps of top 20 up‐and down‐regulated genes in cancer cells (E, F) and proliferating cancer cells (G, H) from TNBC cancer compared to ER+ and HER2+ subtypes. (I–L) Volcano plots of differentially expressed genes between TNBC and ER+/HER2+ subtypes in cancer cells (I) and proliferating cancer cells (L).

Furthermore, we performed comparative analysis of the differentially expressed genes between TNBC versus ER+ and HER2+ subtypes with pseudobulk approach. Multidimensional scaling (MDS) results confirmed the separated distribution among different cell types (Figure 2A,B). We then employed UpSet plots to show the numbers of differentially expressed genes for each cell type and the shared differentially expressed genes between groups (Figure 2C,D). Additionally, the heatmaps revealed the top 20 up‐ and down‐regulated genes between TNBC and ER+ cancer cells (Figure 2E), the top 20 up‐ and down‐regulated genes between TNBC and HER2+ cancer cells (Figure 2F), the top 20 up‐ and down‐regulated genes between TNBC and ER+ proliferating cancer cells (Figure 2F), and the top 20 up‐ and down‐regulated genes between TNBC and HER2+ proliferating cancer cells (Figure 2G). Volcano plots highlighted several significantly upregulated genes in TNBC cancer cells and proliferating cancer cells with the cutoffs of adjusted *p*‐value < 0.05 and |log2FoldChange| > 1.2 (Figure 2I–L).

### 3.3 Common Enriched Pathways Identified in TNBC Compared to ER+ and HER2+ Breast Cancer Subtypes in Cancer Cells

**FIGURE 3:**
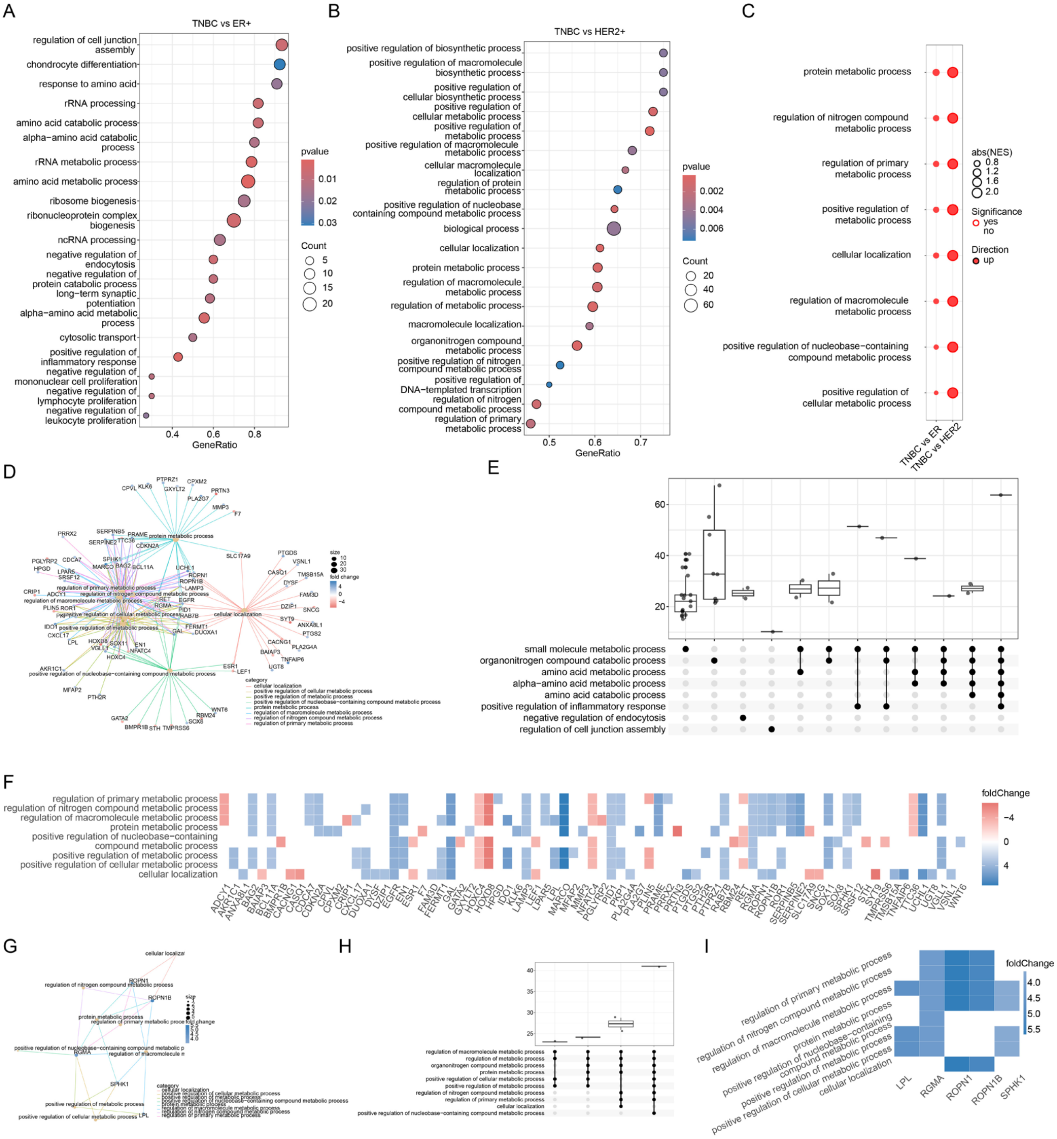
Common enriched pathways identified in TNBC compared to ER+ and HER2+ breast cancer subtypes in cancer cells. (A, B) Top 20 Gene Ontology significantly enriched biological processes identified in TNBC compared to ER+ and HER2+ breast cancer subtypes in cancer cells. Dot size stands for the genes within the pathway, and colour intensity indicates the significance. (C) Eight common enriched pathways in cancer cells between TNBC vs. ER+ and TNBC vs. HER2+ comparisons. (D) Network visualisation of enriched pathways and their relationships in cancer cells between TNBC and ER+ subtype. (E) UpSet plot showing the complex association between the eight common enriched pathways between TNBC and ER+ subtype. (F) Heatmap showing the expression patterns of differentially expressed genes in the eight common enriched pathways between TNBC and ER+ subtype. (G) Network visualisation of enriched pathways and their relationships in cancer cells between TNBC and HER2+ subtype. (H) UpSet plot showing the complex association between the eight common enriched pathways between TNBC and HER2+ subtype. (I) Heatmap showing the expression patterns of differentially expressed genes in the eight common enriched pathways between TNBC and HER2+ subtype.

With the identified differential expressed genes in cancer cells, we then employed gene set enrichment analysis (GSEA) to search for the enriched pathways in each comparison. The dot plots showed the top20 enriched pathways in TNBC cancer cells compared to ER+ and HER2+ cancer cells (Figure 3A,B). Subsequently, we narrowed down the candidate pathways by searching for the consistent pathways between TNBC vs. ER+ and TNBC vs. HER2+ cancer cells, which were predominantly involved in metabolic processes, including protein metabolic, nitrogen compound metabolic, macromolecule metabolic, nucleobase‐containing metabolic, and cellular metabolic processes (Figure 3C). Network analysis revealed the interconnection among these metabolic pathways with protein metabolic process as the central hub in TNBC vs. ER+ cancer cells (Figure 3D) and TNBC vs. HER2+ cancer cells (Figure 3G). The UpSet plots also showed the interconnections between each pathway in TNBC vs. ER+ cancer cells (Figure 3E) and TNBC vs. HER2+ cancer cells (Figure 3H). We also plotted the heatmap for the differential expressed genes in the shared pathways (Figure 3F,I). The altered metabolic pathways were enriched in cancer cells, suggesting the potential correlation between metabolic processes and the enhanced proliferative ability of TNBC cancer cells.

### 3.4 Expression Analysis of Candidate Genes Across Breast Cancer Subtypes

**FIGURE 4:**
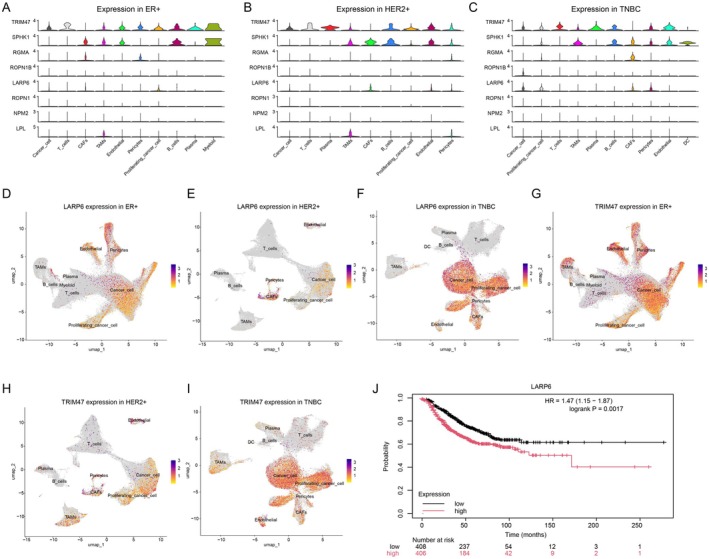
Expression analysis of candidate genes across breast cancer subtypes. (A–C) Stacked violin plots showing expression patterns of the eight common genes within the enriched pathways across different cell types in ER+ (A), HER2+ (B), and TNBC (C) samples. (D–I) UMAP visualisation of LARP6 (D–F) and TRIM47 (G–I) expression patterns across different breast cancer subtypes. (J) Kaplan–Meier survival analysis showing the correlation between LARP6 expression and patient survival (HR = 1.47, *p* = 0.0017).

Subsequently, we further narrowed down the eight candidate genes (TRIM47, SPHK1, RGMA, ROPN1B, LARP6, ROPN1, NPM2 and LPL) that are shared in TNBC compared to ER+ and HER2+ breast cancer subtypes in cancer cells. Stacked violin plots showed the expression levels of these eight common genes in different breast cancer subtypes (Figure 4A–C) and the expression of LARP6 (Figure 4D–F) was significantly up‐regulated in TNBC cancer cells compared to ER+ and HER2+ cancer cells, indicating the potential role of LARP6 in the aggressive behaviours of TNBC cancer cells. TRIM47 (Figure 4G–I) showed a high expression level in all breast cancer subtypes. Interestingly, Kaplan–Meier analysis results showed that high LARP6 levels significantly correlated with poor patient survival (Hazard ratio is 1.47, 95% CI is 1.15–1.87 and logrank *p* = 0.0017) (Figure 4J). The 5 years survival probability for high LARP6 expression patients was significantly lower than the probability for low LARP6 expression patients (Figure 4J). Taken together, our finding demonstrated that LARP6 is a potential key regulator of TNBC progression.

### 3.5 Down‐Regulation of LARP6 Reduced the Cancer Cell Proliferation and Cell Cycle Progression

**FIGURE 5:**
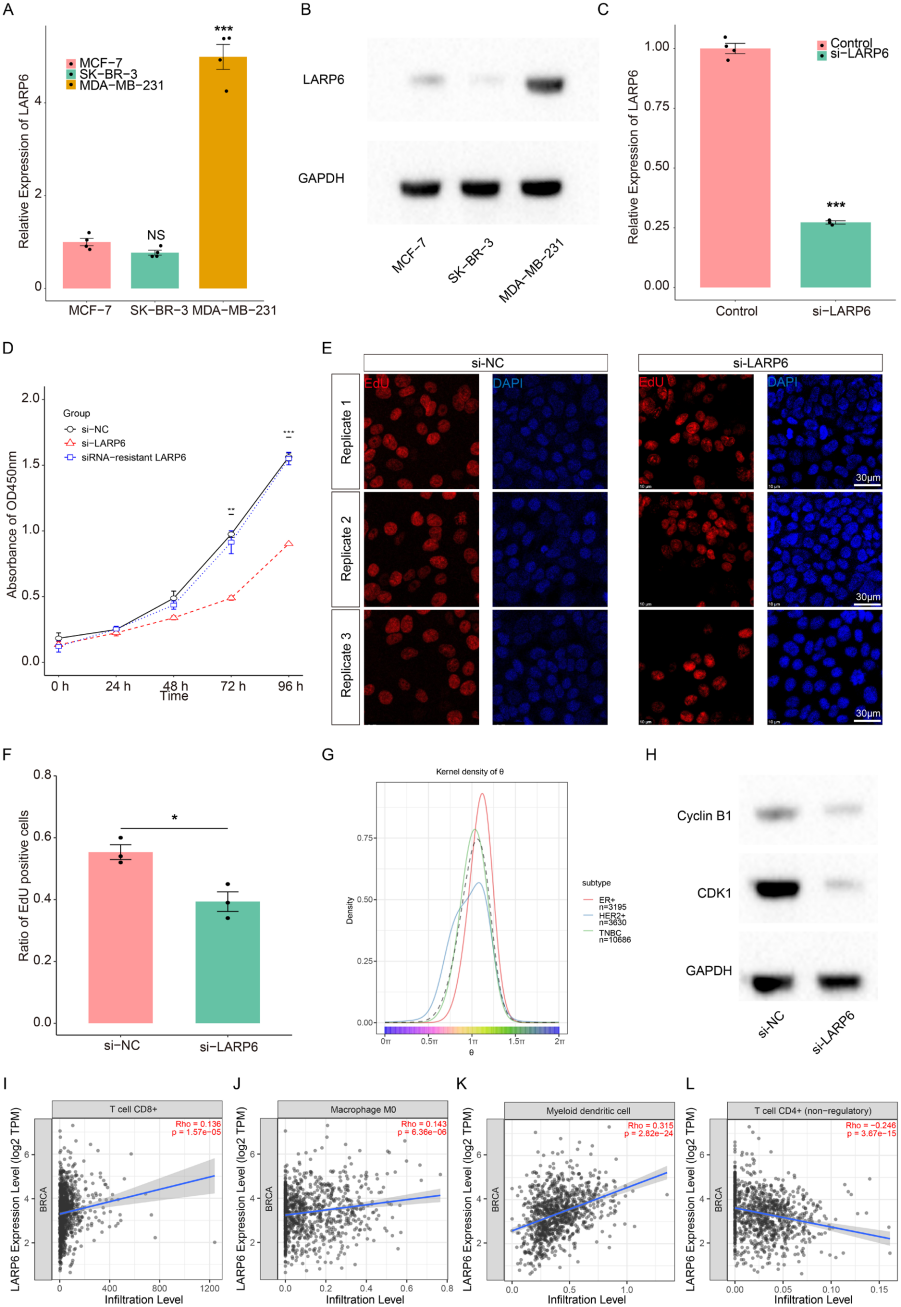
Down‐regulation of LARP6 reduced the cancer cell proliferation and cell cycle progression. (A) qRT‐PCR analysis of LARP6 expressions in ER+, HER2+ and TNBC breast cancer cell lines. (B) Western blot validation of LARP6 protein levels in ER+, HER2+ and TNBC breast cancer cell lines. (C) qRT‐PCR results showing knockdown efficiency of LARP6 siRNA. (D) The Cell Counting Kit‐8 (CCK‐8) assay showing the growth curves of control, LARP6‐knockdown and siRNA‐resistant LARP6 cells. (E) EdU assay results showing the proliferation ability of control and LARP6‐knockdown cells. Scale bar, 10 μm. (F) Quantification of EdU‐positive cells from (E). (G) Cell cycle analysis results for ER+, HER2+ and TNBC subtypes using TriCycle. (H) Western blot analysis showing the Cyclin B1 and CDK1 levels in control and LARP6‐knockdown cells. (I–L) Immune infiltration analysis revealed the correlations between LARP6 expressions and T cell CD8+, Macrophage M0, Myeloid dendritic cell and T cell CD4+ (non‐regulatory). For each experimental condition, we analysed three technical replicates across four independent biological replicates.

Following these observations, we further examined the expression of LARP6 in cell lines from different breast cancer subtypes. Both qRT‐PCR and western blot results confirmed that the LARP6 level was significantly higher in the TNBC cell line MDA‐MB‐231 compared to the ER+ cell line MCF‐7 and HER2+ cell line SK‐BR‐3 (Figure 5A,B). To study the function of LARP6, we employed siRNA‐mediated knockdown, and the qRT‐PCR confirmed the efficiency of siRNA‐mediated knockdown, which was around 75% reduction in the mRNA level (Figure 5C). Additionally, we performed CCK‐8 assay and EdU incorporation assay to explore the role of LARP6 in cancer cell proliferation. CCK‐8 results showed LARP6 knockdown significantly reduced cell proliferation, while proliferation ability was not affected in the siRNA‐resistant LARP6 construct group (Figure 5D). After LARP6 knockdown, the EdU positive cell number was significantly decreased, indicating that LARP6 promoted cell proliferation (Figure 5E,F). We then conducted cell cycle analysis using TriCycle package to explore the cell cycle distribution of the cells at single cell resolution. The result revealed that more proliferating cancer cells from TNBC subtype were distributed at M phase (1.5π is the middle of M phase) (Figure 5G). Consistently, after LARP6 knockdown, western blot results also showed reduced expression of Cyclin B1 and CDK1, which drive the cell cycle progression from G2 to M phase (Figure 5H), indicating that LARP6 promotes the proliferation of TNBC cancer cells via regulation of the cell cycle progression. Our analysis also revealed distinct correlation patterns between LARP6 expressions and various immune cell infiltrates in the breast cancer microenvironment. Specifically, LARP6 expression demonstrated strong positive correlations with CD8+ T cell infiltration, M0 macrophage and myeloid dendritic cell populations, suggesting potential involvement in cytotoxic T cell recruitment and immune cell modulation (Figure 5I–K). Conversely, we observed significant negative correlations with CD4+ non‐regulatory T cells (Figure 5L). These findings suggest that LARP6 may play a dual role in breast cancer progression—directly promoting cancer cell proliferation through cell cycle regulation as demonstrated in our functional studies, while simultaneously modulating the tumour immune microenvironment. The positive correlation with CD8+ T cells indicates that LARP6 might paradoxically enhance anti‐tumour immunity through cytotoxic T cell recruitment, potentially representing a compensatory mechanism activated in response to aggressive tumour growth. Similarly, the positive association with myeloid dendritic cells suggests potential enhancement of antigen presentation, though the functional significance of this correlation requires further investigation. The relationship with undifferentiated M0 macrophages and negative correlation with CD4+ non‐regulatory T cells points to a complex immunomodulatory role for LARP6 in shaping the breast cancer microenvironment that warrants further mechanistic studies.

## 4 Discussion

In this study, we employed the integration of extensive single‐cell RNA sequencing data to uncover new therapeutic targets within triple‐negative breast cancer (TNBC). Our thorough RNA‐seq analyses conducted on more than 260,000 cells across various breast cancer subtypes demonstrated that LARP6 expression is notably increased in TNBC compared to ER+ and HER2+ variants, highlighting LARP6 as a potential key regulator of TNBC progression. Furthermore, survival analysis suggested that patients exhibiting high levels of LARP6 are more likely to experience unfavourable outcomes, underscoring its clinical relevance. Significantly, our functional assays indicated that depleting LARP6 markedly inhibits the proliferation of TNBC cells through alterations in cell cycle dynamics. Based on the immune cell infiltration analysis, LARP6 simultaneously modulates the tumour immune microenvironment in breast cancers.

It is an important step towards the integration and analysis of single‐cell RNA sequencing data across different breast cancer subtypes as compared with conventional bulk sequencing approaches [19, 25]. This approach allowed the determination of cellular heterogeneity within different breast cancer subtypes as well as revealing cell type‐specific gene expression signatures otherwise hidden in bulk analysis. Using pseudobulk analysis, we could robustly ascertain differentially expressed genes while accounting for inter‐patient variability. As such, this approach was especially potent in identifying TNBC‐specific markers since it enabled us to investigate gene expression patterns within different cellular populations comprising the tumour microenvironment.

Mechanistically, our results indicate that LARP6 may drive TNBC progression via modulation of cell cycle‐related processes. Although LARP6 was previously suggested to be involved in RNA metabolism and cellular homeostasis [17, 26, 27, 28, 29], a role for LARP6 in TNBC has not been defined prior to the current studies. These phenotypes correlate with reported action of other LARP family members in cancer and imply an evolutionarily conserved role in growth and survival in the cancer context. However, the upregulation of LARP6 in TNBC suggests that it has subtype‐specific roles in this breast cancer variant.

Given that the current lack of targetable therapies for TNBC subtype [9, 30, 31], the clinical implications of our finding are particularly significant. The survival analysis suggests that LARP6 could be a prognostic marker and help to identify aggressive TNBC patients, who could be given more aggressive treatment. The potential of LARP6 as a potential TNBC specific marker opens new avenues for clinical management of TNBC patients. LARP6, an RNA‐binding protein, is a potential molecular target, and the development of novel specific inhibitors for LARP6 could be a novel treatment strategy for TNBC patients. Future studies incorporating ChIP‐seq, ATAC‐seq, and transcription factor knockdown experiments across multiple cell lines representing each breast cancer subtype would provide mechanistic insights into the differential regulation of LARP6 expression. Understanding these subtype‐specific regulatory mechanisms could reveal additional therapeutic vulnerabilities in TNBC. Identification of specific RNA targets bound by LARP6 in TNBC could catalyse multiple therapeutic strategies. First, elucidation of the downstream mRNA targets regulated by LARP6 would reveal secondary therapeutic targets that may be more druggable than RNA‐binding proteins themselves. Second, LARP6‐dependent RNA signatures could serve as biomarkers for patient stratification, identifying those most likely to benefit from LARP6‐targeted interventions. These approaches collectively represent promising avenues to address the significant unmet clinical need in TNBC treatment.

Even these results are promising, some limitations and future study directions should be noted. Firstly, our in vitro studies suggest LARP6 functions in regulating the proliferation of the TNBC cancer cells; the in vivo validation of the LARP6 role will be necessary to investigate the therapeutic potential of targeting LARP6. Second, the molecular downstream mechanisms by which LARP6 regulates cell cycle progression remain unknown. Future studies should explore the RNA targets of LARP6 in the TNBC subtype.

In conclusion, our study reveals LARP6 as an important regulator of TNBC cancer cell proliferation by comprehensive integration of single‐cell RNA sequencing datasets and following differential gene expression analysis and pathway enrichment analysis. The high expression level of LARP6 in the TNBC subtype compared to ER+ and HER2+ subtypes, which is correlated with a poor breast cancer survival rate, highlights its potential to be a TNBC specific prognostic marker and a TNBC specific therapeutic target. Our findings advance our understanding of TNBC progression and provide a potential therapy strategy by targeting the novel druggable molecular target. Future studies about the downstream molecular mechanisms and in vivo phenotype confirmation will be essential for the translation of our findings into clinical management strategies for TNBC patients.

## Author Contributions

**Feng Yuan:** data curation (lead), formal analysis (lead), investigation (lead), software (lead), validation (lead), visualization (lead), writing – original draft (supporting). **Gai Liang:** data curation (supporting), formal analysis (supporting), investigation (supporting), methodology (supporting). **Qu Zhang:** data curation (supporting), formal analysis (supporting), investigation (supporting), methodology (supporting). **Bo Luo:** data curation (supporting), formal analysis (supporting), investigation (supporting), methodology (supporting). **Jianhua Liu:** data curation (supporting), formal analysis (supporting), investigation (supporting), methodology (supporting). **Xinhong Wu:** conceptualization (lead), funding acquisition (lead), project administration (lead), resources (lead), supervision (lead), writing – original draft (lead), writing – review and editing (lead).

## Conflicts of Interest

The authors declare no conflicts of interest.

## Data Availability

The data supporting the findings within this study are presented in the main text and figures.
